# Diet, Nutrition, Obesity, and Their Implications for COVID-19 Mortality: Development of a Marginalized Two-Part Model for Semicontinuous Data

**DOI:** 10.2196/22717

**Published:** 2021-01-26

**Authors:** Naser Kamyari, Ali Reza Soltanian, Hossein Mahjub, Abbas Moghimbeigi

**Affiliations:** 1 Department of Biostatistics School of Public Health Hamadan University of Medical Sciences Hamadan Iran; 2 Modeling of Noncommunicable Diseases Research Center Hamadan University of Medical Sciences Hamadan Iran; 3 Research Center for Health Sciences School of Public Health Hamadan University of Medical Sciences Hamadan Iran; 4 Department of Biostatistics and Epidemiology School of Health & Determinants of Health Research Center Alborz University of Medical Sciences Karaj Iran

**Keywords:** COVID-19, diet, nutrition, obesity, marginalized two-part model, semicontinuous data

## Abstract

**Background:**

Nutrition is not a treatment for COVID-19, but it is a modifiable contributor to the development of chronic disease, which is highly associated with COVID-19 severe illness and deaths. A well-balanced diet and healthy patterns of eating strengthen the immune system, improve immunometabolism, and reduce the risk of chronic disease and infectious diseases.

**Objective:**

This study aims to assess the effect of diet, nutrition, obesity, and their implications for COVID-19 mortality among 188 countries by using new statistical marginalized two-part models.

**Methods:**

We globally evaluated the distribution of diet and nutrition at the national level while considering the variations between different World Health Organization regions. The effects of food supply categories and obesity on (as well as associations with) the number of deaths and the number of recoveries were reported globally by estimating coefficients and conducting color maps.

**Results:**

The findings show that a 1% increase in supplementation of pulses reduced the odds of having a zero death by 4-fold (OR 4.12, 95% CI 11.97-1.42). In addition, a 1% increase in supplementation of animal products and meat increased the odds of having a zero death by 1.076-fold (OR 1.076, 95% CI 1.01-1.15) and 1.13-fold (OR 1.13, 95% CI 1.0-1.28), respectively. Tree nuts reduced the odds of having a zero death, and vegetables increased the number of deaths. Globally, the results also showed that populations (countries) who consume more eggs, cereals excluding beer, spices, and stimulants had the greatest impact on the recovery of patients with COVID-19. In addition, populations that consume more meat, vegetal products, sugar and sweeteners, sugar crops, animal fats, and animal products were associated with more death and less recoveries in patients. The effect of consuming sugar products on mortality was considerable, and obesity has affected increased death rates and reduced recovery rates.

**Conclusions:**

Although there are differences in dietary patterns, overall, unbalanced diets are a health threat across the world and not only affect death rates but also the quality of life. To achieve the best results in preventing nutrition-related pandemic diseases, strategies and policies should fully recognize the essential role of both diet and obesity in determining good nutrition and optimal health. Policies and programs must address the need for change at the individual level and make modifications in society and the environment to make healthier choices accessible and preferable.

## Introduction

Transmission of COVID-19 began in Wuhan, Hubei Province, China on December 31, 2019 [1,2]. According to the latest World Health Organization (WHO) report on July 3, 2020, there were 11,188,120 confirmed cases and 528,431 deaths worldwide, with 1505 total cases and 69.3 deaths per 1 million population [3]. The WHO named it a global pandemic because of the rapid outbreak of the disease worldwide [4,5].

The COVID-19 epidemic started during winter in areas of the world where the consumption of wildlife is not uncommon. Coronavirus is one of the viruses causing the common cold, a disease that has never had a cure nor any effective prevention or vaccine. However, there are relatively consistent data suggesting that the risk of contracting the common cold is high under inadequate sleep, psychosocial or physical stress including exposure to cold temperatures, inadequate nutrition, and any condition that compromises the body’s immune system [6].

A high percentage of COVID-19 deaths worldwide are associated with one or more chronic conditions. It is also evident that older people are at a higher risk for severe illness with this pandemic [7,8]. Nutrition is not a treatment for COVID-19, but it is a modifiable contributor to the development of chronic disease, which is highly associated with COVID-19 severe illness and deaths [9]. A well-balanced diet and healthy patterns of eating strengthens the immune system, improves immunometabolism, and reduces the risk of chronic disease and infectious diseases [10,11]. Furthermore, nutrition may have a positive or negative impact on COVID-19, as it may be a way to support people at higher risk for the disease (ie, older people and people with pre-existing conditions of noncommunicable diseases) [12].

It is clear in these challenging times that optimizing nutrition is important, not only for ourselves but also for every patient that goes through their own period of treatment. Every health system should be aware of the benefits of healthy eating and be able to provide sound nutritional guidance to their patients, especially those with chronic disease. Having knowledge about nutritional interventions that may help prevent chronic conditions and their associated risks is now more important than ever [13].

On the other hand, being overweight or obese are interpreted as excessive fat [14] accumulation and represent a risk to health [15]. Most of the world’s populations live in countries where being overweight or obese kill more people than being underweight. However, does it cause a decrease in the immune system or severity of COVID-19? Is it dangerous toward getting an infection and the mortality of COVID-19?

This study aims to assess the effect of diet, nutrition, and obesity on COVID-19 mortality among 188 countries by using new statistical marginalized two-part (mTP) models. Hence, we globally evaluated the distribution of diet and nutrition on the national level while considering the variation between different regions. The effects of food supply categories and obesities on (as well as associations with) the number of deaths and the number of recoveries is reported worldwide by estimating coefficients and conducting color maps.

## Methods

### Overview

This section starts with a short description of the data set and information on relevant sources. In the following section, we introduce the conventional two-part (TP) regression model and the proposed mTP regression model for semicontinuous data. For the continuous part, we considered two flexible distributions including log-normal (LN) and beta prime (BP). We also described their properties to assess the overall impact of covariates on the marginal mean and demonstrated that the proposed model outperforms the conventional model. Finally, the proposed mTP model was applied to the healthy diet data set on fat quantity and protein to investigate the effects of nutrition categories and obesity on the number of deaths and recoveries in 100 cases of COVID-19.

### Dietary, Obesity, and COVID-19 Data

Food supply data is some of the most important data in both Food and Agriculture Organization (FAO)/WHO STAT [16]. In fact, this data is the basis for estimations of global and national undernourishment assessment when it is combined with parameters and other data sets. In addition, both businesses and governments use this data for economic analysis and policy setting, and the academic community also uses this data.

**Figure 1:**
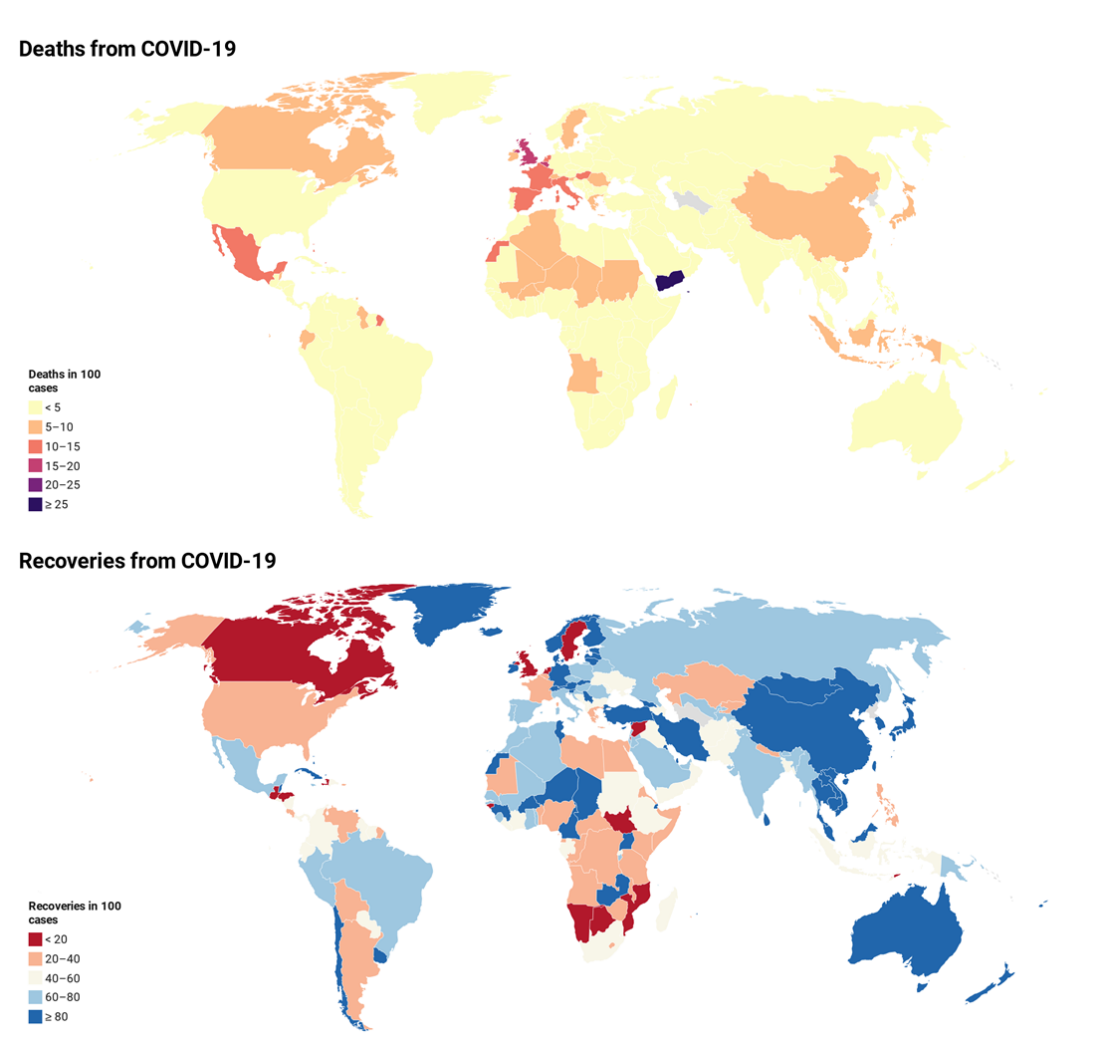
World map related to the number of deaths (top) and the number of recoveries (bottom) in 100 cases of COVID-19 as of July 3, 2020.

In this data set, we combined data of different types of food, world population obesity and undernourished rates, and the global COVID-19 cases count from around the world (188 countries) to learn more about how a healthy eating style could help combat COVID-19. In addition, from the data set, we can gather information regarding diet patterns from countries with lower COVID-19 infection rates and adjust our own diet accordingly. The spread of the disease, deaths, recoveries, and their different distributions are shown in Figure 1, which can be evaluated according to the WHO regions.

From the data sets, accessible as Google sheets in GitHub [17], we have used fat quantity and protein for different categories of food (all calculated as percentage of total intake amount). We have also added on the obesity rate (in percentage) for comparison. The end of the data sets also included the most up-to-date confirmed infections, deaths, recoveries, and active cases (also in percentage of current population for each country). In this study, response variables were the deaths in 100 cases and the recoveries in 100 cases that were continuously (ranged 0 to 100) measured for 188 countries [18].

To synchronize results relative to interregional variations, data sets were grouped according to WHO regions (Multimedia Appendix 1), and a mTP analysis of deaths and recoveries was conducted using a random effects (regions cluster) model. Supply food data description is described in Multimedia Appendix 2. Both fat quantity and protein data sets, including 23 categories, were obtained from the FAO database [19] and were used to show the specific types of food that belong to each category for assessing influential effects of the fat quantity and protein supply.

Semicontinuous response variables such as mortality indexes are typically characterized by the presence of zeros and positive continuous outcomes that are often right skewed. In this paper, we propose a class of models for positive and zero responses by means of a zero-augmented mixed regression model. Under this class, we are particularly interested in studying positive responses whose distribution accommodates skewness. At the same time, responses can be zero, and therefore, we justified the use of a zero-augmented mixture model.

### Marginalized Two-Part Models for Semicontinuous Data

#### Conventional Two-Part Model

We began with a review of the conventional TP model presented in Cragg [20], Manning et al [21], Duan et al [22], and elsewhere. Let *Y_ij_* be a semicontinuous variable for the i-*th* (*i*=1,2, ..., *n*) subject at cluster j (*j*=1,2, ..., *n_i_*). For nonnegative data (*Y_ij_*≥0) consisting of independent observations that clustered in the j-*th* level, the generic form of the conventional TP model can be written as:

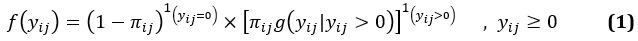



where *π_ij_* = *Pr*(*Y_ij_*>0), 1_(.)_ is the indicator function, and *g*(*y_ij_*|*y_ij_*>0) is any density function applicable to the positive values of *Y_ij_*, although the LN density is often chosen. This model is parameterized following equations (2) and (3) relevant to zero and nonzero components, respectively:

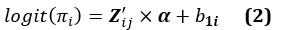



where ***Z****_ij_* is a 1 × *q* covariate (used as an explanatory variable) vector, ***α*** is a *q* × 1 regression coefficient vector, and *b***_1_*_i_*** is the cluster-level random effect in the zero component. The location parameter *μ_ij_* is modeled in the second part of the TP model assuming a log link:

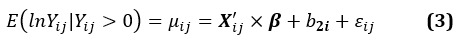



where ***X****_ij_* is a 1 × *p* covariate vector, ***β*** is a *p* × 1 regression coefficient vector, and *b***_2_*_i_*** is again the cluster-level random effect in the nonzero component. The error term *ε_ij_* is assumed to be normally distributed as *N*(0, 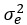). Note that this TP mixed model can be extended to include additional random effects. For illustration purposes and simplicity, we restricted attention here to the TP mixed models with two levels; extensions to multilevel models are straightforward.

When fitting this model to independent responses, the binary and conditionally continuous components of the likelihood are separable, and therefore, these two parts are fit separately. The binary component is often modeled using logistic regression, and the continuous component can be fit using standard regression models such as the BP [23], LN [24], gamma [25,26], and long-skew normal [27].

The marginal mean and variance of *Y_ij_* from a TP model can be derived as follows:

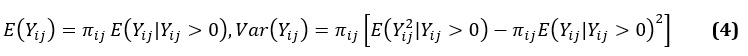



When LN is assumed in the continuous part, the marginal mean is:

*E*(*Y_ij_*) = *ν_ij_* = *π_ij_* *exp*{*μ_ij_* + *σ*^2^ ⁄ 2} **(5)**

#### Marginalized Two-Part Model

To obtain interpretable covariate effects on the marginal (unconditional) mean, we proposed the following mTP model that parameterizes the covariate effects directly in terms of the marginal mean, *ν_i_*=*E*(Y_i_), on the original (ie, untransformed) data scale. The mTP model specifies the linear predictors:

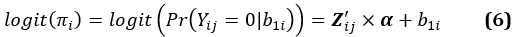



where *b*_1_*_i_* represents the random effect that accounts for the within-subject correlation pertaining to the clustered measures in the zero part, *b*_1_*_i_* ~ *N*(0, 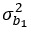).

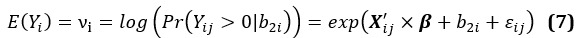



where *b*_2_*_i_* represents the random effect that accounts for the within-subject correlation pertaining to the clustered measures in the continuous part, *b*_2_*_i_* ~ *N*(0, 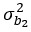).

The two random effect intercepts *b*_1_*_i_* and *b*_2_*_i_* in the two processes of zero and nonzero are assumed to be independent and uncorrelated. 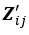 is the vector of covariates for the i-*th* subject measured at the j-*th* cluster for the binary part, and 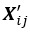 is the vector of covariates for the i-*th* subject measured at the j-*th* cluster used for the continuous part. The two parts might have common covariates or completely different ones. ***α*** is the vector of model coefficients corresponding to the binary part, and ***β*** is the vector of coefficients corresponding to the continuous part, conditional on the values being nonzero. The model can be easily extended to include higher-order random effects.

#### Marginalized Two-Part Log-Normal Model

When modeling semicontinuous data, the continuous component is most frequently modeled using a LN distribution. The generic form of the marginalized two-part log-normal (mTP LN) model for independent responses can be written as in equation (1), with *g*(*y_ij_*|*y_ij_*>0), taking the LN density function *LN*(.; *μ*, *σ*^2^) with mean *μ* and variance *σ*^2^ on the log scale. The marginal mean and variance of *Y_ij_* are:

*E*(*Y_ij_*) = *ν_ij_* = *π_ij_exp*(*μ_ij_* + *σ*^2^ ⁄ 2) **(8)**

*Var*(*Y_ij_*) = *π_ij_exp*(2*μ_ij_* + *σ*^2^)[*exp*(*σ*^2^) – *π_ij_*] **(9)**

The likelihood (*L*), parameterized in terms of *π_ij_* and *μ_ij_*, is:

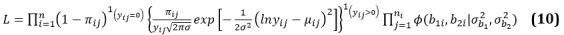



where *ϕ*(*b*_1_*_ij_*, *b*_2_*_ij_*) represents the bivariate normal distribution for the random effects with a mean vector of zeros and variance-covariance matrix 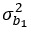 and 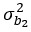 for zero and nonzero parts, respectively.

To use this LN likelihood framework, the marginal mean in equation (8) can be rearranged to solve for *μ_ij_*, yielding:

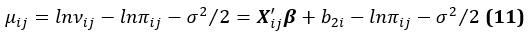



Noting that:

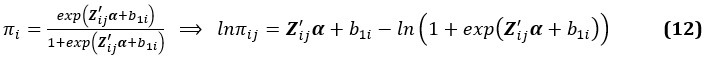



and:

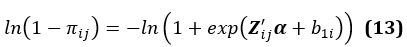



#### Marginalized Two-Part Beta Prime Model

The BP distribution [28,29] is also known as inverted beta distribution or beta distribution of the second kind, often the model of choice for fitting semicontinuous data where the response variable is measured continuously on the positive real line (*Y*>0) because of the flexibility it provides in terms of the variety of shapes it can accommodate. The probability density function of a BP distributed random variable *Y* parameterized in terms of its mean *μ* and a precision parameter *ϕ* is given by:

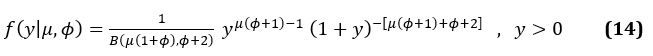



where, *B* denotes the beta function *μ*>0, *ϕ*>0, *E*(*Y*) = *μ*, and *Var*(*Y*) = (*μ*(1 + *μ*)) / *ϕ*.

To obtain interpretable covariate effects on the marginal mean, we proposed the following mTP model that parameterizes the covariate effects directly in terms of the marginal mean, ν_ij_ = E(Y_ij_), on the original (ie, untransformed) data scale. The mTP model with random (cluster) effects ***Z***_1_*_ij_* and ***Z***_2_*_ij_* for the zero and the continuous components, respectively, specifies the linear predictors:

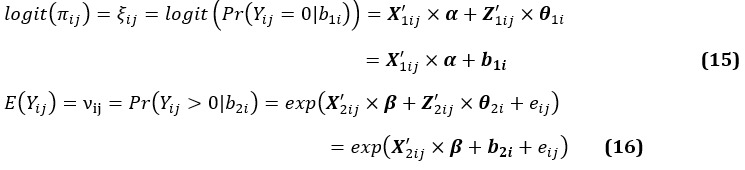



where, 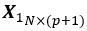 and 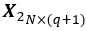 have full rank *p* and *q* for the zero and the continuous components, respectively; ***α***_(_*_p_*_+1)×1_ and ***β***_(_*_q_*_+1)×1_ are the corresponding vectors of the regression coefficients. As seen in equations (15) and (16), the mixing probability and mean of the component of the continuous parts are linked to the independent variables through logit and logarithmic link functions. The vectors ***b***_1_=(*b*_11_, *b*_12_, ..., *b*_1_*_m_*)' and ***b***_2_=(*b*_21_, *b*_22_, ..., *b*_2_*_m_*)' denote random effects of the third level in the components of logistic and continuous, respectively. For simplicity of interpretation and mathematical calculations, the random effects (*b*_1_, *b*_2_) were assumed to be joint normally distributed with mean zero and variances 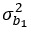 and 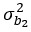, respectively [30,31]. The errors term *e_ij_* ~ *N*(0, 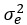) was also assumed to be of normal distribution and independent of the random effects.

Let *ψ_ij_* = *I*(*y_ij_*>0) denote the indicator of *Y_ij_* being nonzero. The general form of the likelihood function for the i-*th* subject can be described as follows:

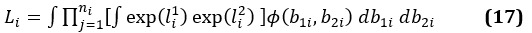



where the log-likelihood for the binary part is:

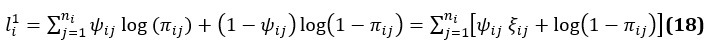



and the log-likelihood for the continuous part is:

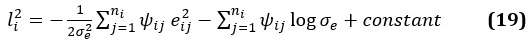



with 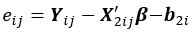, which can be implemented in the SAS NLMIXED procedure by quasi-Newton optimization with adaptive Gaussian quadrature techniques [32]. With the conventional model, the likelihood and score equations can be separated into two independent components: one for the binary part and one for the continuous part. In contrast, note that the score equations for the mTP model are not separable, and thus, the binary and continuous parts are fit simultaneously. Model-based asymptotic standard errors are computed using Fisher information matrix, Ι(***α***, ***β***, *σ*), as:

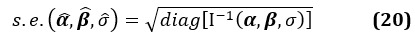



with the maximum likelihood estimates substituted for ***α***, ***β***, and *σ*.

## Results

In this section, the proposed mTP model was applied to the healthy diet data set on fat and protein to investigate the effects of supplementation categories on the number of deaths per 100 cases and recoveries per 100 cases of COVID-19. The estimations of mTP BP and mTP LN related to deaths and recoveries are shown in Tables 1 and 2, respectively. In these tables, variances (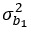 and 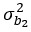) show the variety of responses among level 2 (ie, the WHO regions) related to each part of the zero and nonzero (ie, positive) components. Tables 1 and 2 show that almost all categories have the same effect on the number of deaths and recoveries in 100 cases. The number of deaths per 100 cases, number of recoveries per 100 cases, and the obesity rates until July 3, 2020, for all countries and split by the WHO regions is shown in Multimedia Appendix 3. Deaths are more common in Western and Southwest Europe (eg, Belgium, the United Kingdom, France, Italy, Hungary, Netherlands, and Spain), North America (eg, Mexico, Bahamas, Canada, Barbados, Belize, and the United States), and North Africa (eg, Western Sahara, Chad, Algeria, and Niger). The highest number of deaths occurred in Yemen (26.62 deaths per 100 cases), which could be due to the crises caused by the war and the poor health conditions in this country in the last years. Frequently, it seems that the northern regions of the world appear to have had more deaths, which may be due to temperature differences between the two hemispheres.

**Table 1:** Results of marginalized two-part BP and LN model in predicting number of deaths per 100 cases and considering the cluster effect of World Health Organization regions in the fat data set.

Fat (categories)		Fat quantity				Protein			
		Zero component		Nonzero component		Zero component		Nonzero component	
		Coefficient (α)	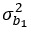	Coefficient (β)	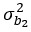	Coefficient (α)	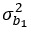	Coefficient (β)	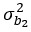
**Alcoholic beverages**									
	BP^a^	—^b^	—	–7.0449	0.1145	1.3670	0.4907	0.04707	0.1286
	LN^c^	—	—	–6.4512	0.1165	1.3752	0.4814	0.04918	0.1189
**Animal products**									
	BP	0.0401	0.7457	–0.0028	0.1539	*0.0736* ^d^	0.6212	0.0105	0.1236
	LN	0.0405	0.6543	–0.0031	0.1495	*0.0812*	0.5965	0.0108	0.1209
**Animal fats**									
	BP	0.1505	0.6458	0.0009	0.1223	7.6487	0.3055	0.3259	0.1212
	LN	0.1489	0.6147	0.0009	0.1239	7.5461	0.2891	0.3319	0.1801
**Aquatic products other**									
	BP	4.3474	0.7746	–13.9857	0.1123	30.9618	0.4060	–0.7270	0.1201
	LN	4.5421	0.7469	–10.2576	0.1127	29.1456	0.3995	–0.7345	0.1193
**Cereals excluding beer**									
	BP	–0.0839	0.6195	–0.0218	0.1485	–0.0548	0.4994	–0.0171	0.1163
	LN	–0.0956	0.6015	–0.0221	0.1399	–0.0551	0.4861	–0.0170	0.1123
**Eggs**									
	BP	0.3217	0.6155	–0.1291	0.1391	0.3993	0.4487	–0.0466	0.1297
	LN	0.3514	0.5412	–0.1299	0.1381	0.4125	0.4912	–0.0481	0.1183
**Fish seafood**									
	BP	–0.0556	0.6000	0.0383	0.1903	0.0542	0.4220	0.0305	01318
	LN	–0.0598	0.5816	0.0401	0.1849	0.0556	0.4001	0.0351	0.1301
**Fruits excluding wine**									
	BP	–0.4420	0.6323	0.0933	0.1633	–0.4744	0.5036	0.0783	0.1176
	LN	–0.4511	0.5971	0.0931	0.1617	–0.4598	0.5121	0.0803	0.1165
**Meat**									
	BP	0.0394	0.7340	0.0008	0.1699	*0.1246*	0.4690	0.0256	0.1174
	LN	0.0391	0.7300	0.0008	0.1684	*0.1268*	0.4581	0.0221	0.1173
**Miscellaneous**									
	BP	2.9863	0.6822	2.0655	0.0994	0.0900	0.6768	–0.0090	0.1336
	LN	2.5531	0.5836	2.1510	0.0991	0.0912	0.6154	–0.0061	0.1136
**Milk excluding butter**									
	BP	0.0589	0.6160	–0.0139	0.1667	0.5456	0.4718	0.0561	0.1259
	LN	0.0512	0.6013	–0.0142	0.1561	0.5537	0.4316	0.0560	0.1241
**Offals**									
	BP	0.0196	0.6429	–0.1844	0.1022	–0.0526	0.4808	0.0704	0.1227
	LN	0.0197	0.6129	–0.1798	0.1124	–0.0541	0.4493	0.0713	0.1201
**Oilcrops**									
	BP	–0.2833	0.5580	0.0179	0.1876	–0.1453	0.5600	0.0090	0.1245
	LN	–0.2891	0.5137	0.0184	0.1773	–0.1457	0.5413	0.0094	0.1239
**Pulses**									
	BP	*–1.4170*	0.1944	0.1150	0.1434	–0.1583	0.4308	0.1496	0.1290
	LN	*–1.4242*	0.1832	0.1159	0.1400	–0.1581	0.4311	0.1499	0.1289
**Spices**									
	BP	–0.5889	0.4622	0.0476	0.1907	–0.0916	0.4309	–0.0561	0.1161
	LN	–0.5887	0.4604	0.0467	0.1891	–0.0914	0.4312	–0.0551	0.1163
**Starchy roots**									
	BP	–0.9187	0.5559	–0.3262	0.1637	0.3208	0.4513	–0.0469	0.1246
	LN	–0.9188	0.5412	–0.3301	0.1621	0.3208	0.4511	–0.0461	0.1212
**Stimulants**									
	BP	0.6042	0.6715	–0.0287	0.1704	0.3476	0.4530	–2.4158	0.1227
	LN	0.6101	0.6712	–0.0288	0.1698	0.3479	0.4530	–2.4129	0.1201
**Sugar crops**									
	BP	–13.0537	0.5727	–6.2283	0.1063	5.7548	0.4394	1.4861	0.1217
	LN	–12.5132	0.5624	–6.3120	0.1059	5.5431	0.4329	1.4869	0.1214
**Sugar sweeteners**									
	BP	–0.8040	0.6104	10.2992	0.2136	1.7184	0.4899	0.5042	0.1328
	LN	–0.8042	0.6112	9.4532	0.2013	1.7204	0.4782	0.5100	0.1236
**Tree nuts**									
	BP	0.3739	0.6207	0.1138	0.2178	*–0.0736*	0.6211	–0.0105	0.1236
	LN	0.3721	0.6211	0.1142	0.2017	*–0.0740*	0.6127	–0.0104	0.1221
**Vegetal products**									
	BP	–0.0401	0.8313	0.0028	0.1485	39.1538	0.5764	2.8053	0.1327
	LN	–0.0413	0.8219	0.0027	0.1449	27.1870	0.4365	2.8101	0.1254
**Vegetable oils**									
	BP	–0.0008	0.6667	0.0025	0.1663	0.0228	0.4442	–0.0376	0.1228
	LN	–0.0012	0.6120	0.0024	0.1659	0.0224	0.4318	–0.0354	0.1224
**Vegetables**									
	BP	–0.5748	0.6205	–0.4784	0.1099	1.3637	0.3991	*0.7713*	0.1050
	LN	–0.5739	0.5945	–0.4754	0.1098	1.3641	0.3981	*0.7716*	0.1002
**Obesity**									
	BP	0.0228	0.5954	0.0054	0.1716	0.0228	0.4798	0.0054	0.1261
	LN	0.0212	0.5871	0.0057	0.1624	0.0227	0.4821	0.0034	0.1178

^a^BP: beta prime.

^b^Empty cells related to unestimated or nonconverged values.

^c^LN: log-normal.

^d^Italics indicate statistical significance at the .05 significance level.

**Table 2:** Results of marginalized two-part BP and LN model in predicting number of deaths in 100 cases and considering the cluster effect of World Health Organization regions in the protein data set.

Protein (categories)		Fat quantity				Protein			
		Zero component		Nonzero component		Zero component		Nonzero component	
		Coefficient (α)	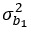	Coefficient (β)	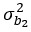	Coefficient (α)	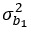	Coefficient (β)	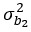
**Alcoholic beverages**									
	BP^a^	0.3934	—^b^	–3.3321	0.0053	–0.5040	—	0.3291	0.0241
	LN^c^	0.3941	0.6541	–3.3520	0.0051	-0.5139	—	0.3301	0.0240
**Animal products**									
	BP	–0.0518	—	–0.0030	5.0390	–0.0377	—	–0.0036	—
	LN	–0.0520	—	–0.0031	5.0011	–0.0378	—	–0.0035	—
**Animal fats**									
	BP	–0.1417	0.6458	0.0037	0.1223	*–4.3929* ^d^	0.6854	–0.1882	0.0291
	LN	–0.1419	0.5454	0.0039	0.1221	*–4.3821*	0.6855	–0.1881	0.0297
**Aquatic products other**									
	BP	0.1026	0.7746	0.0998	0.1123	13.6501	0.6543	–0.1588	0.0102
	LN	0.9817	0.7751	0.0981	0.1123	11.5479	0.6571	–0.1581	0.0115
**Cereals excluding beer**									
	BP	0.2108	0.6195	0.0201	0.1485	0.0935	0.5987	0.0070	—
	LN	0.2107	0.5981	0.0200	0.1498	0.0992	0.4564	0.0074	0.0120
**Eggs**									
	BP	1.0198	0.6155	0.0585	0.1391	0.3015	0.7154	0.0971	1.0199
	LN	1.0211	0.6154	0.0594	0.1384	0.3101	0.6245	0.0954	0.8745
**Fish seafood**									
	BP	2.1882	0.6000	–0.0587	0.1903	0.3784	0.6542	–0.0109	2.0553
	LN	2.1452	0.6124	–0.0591	0.1914	0.3781	0.6549	–0.0117	2.4923
**Fruits excluding wine**									
	BP	3.5529	0.6323	0.0049	0.1633	0.5023	0.6980	–0.0284	0.3869
	LN	3.5504	0.6341	0.0051	0.1601	0.5001	0.7401	–0.0294	0.3881
**Meat**									
	BP	–0.0020	0.7340	–0.0081	0.1699	–0.0720	0.7012	–0.0050	2.8965
	LN	–0.0019	0.7341	–0.0078	0.1692	–0.0711	0.6984	–0.0050	2.8457
**Miscellaneous**									
	BP	8.988	0.6822	–0.1517	0.0994	–0.1208	0.6503	–0.0057	1.8106
	LN	7.5415	0.6824	–0.1540	0.0982	–0.1235	0.6511	–0.0064	1.8001
**Milk excluding butter**									
	BP	–0.1359	0.6160	–0.0020	0.1667	–0.3806	0.4562	0.0398	0.4047
	LN	–0.1314	0.5912	–0.0080	0.1724	–0.3817	0.4575	0.0410	0.4521
**Offals**									
	BP	–3.0231	0.6429	–0.1611	0.1022	–0.1169	0.6985	0.0301	—
	LN	–3.0024	0.6540	–0.1617	0.1512	–0.1141	0.7211	0.0341	—
**Oilcrops**									
	BP	0.0091	0.5580	0.0070	0.1876	–0.1958	0.5913	–0.0269	1.2942
	LN	0.0084	0.5557	0.0072	0.1868	–0.1954	0.5914	–0.0264	1.3125
**Pulses**									
	BP	*–1.6086*	0.1944	–0.3639	0.1434	4.1312	0.2456	–0.2215	0.0303
	LN	*–1.5401*	0.1984	–0.3578	0.1545	4.0128	0.2541	–0.2211	0.0311
**Spices**									
	BP	3.1903	0.4622	–0.2388	0.1907	0.0560	0.5441	0.0208	0.6601
	LN	3.1912	0.4684	–0.2491	0.1912	0.0541	0.5237	0.0214	0.6641
**Starchy roots**									
	BP	0.4714	0.5559	0.0997	0.1637	–0.2431	0.6003	0.1089	0.0603
	LN	0.4787	0.5651	0.0984	0.1746	–0.2433	0.6210	0.1146	0.0701
**Stimulants**									
	BP	0.5780	0.6715	0.1503	0.1704	–0.0752	0.6439	2.1776	0.0100
	LN	0.5417	0.6871	0.1511	0.1724	–0.754	0.7431	2.1290	0.0150
**Sugar crops**									
	BP	0.3393	0.5727	0.9821	0.1063	0.3819	0.5589	*–4.7273*	0.1670
	LN	0.3365	0.5821	0.9807	0.1163	0.3814	0.5613	*–4.7198*	0.1687
**Sugar sweeteners**									
	BP	1.3970	0.6104	*–9.6769*	0.2136	0.5242	0.6987	–0.4085	0.2326
	LN	1.4121	0.6255	*–9.5421*	0.2166	0.5420	0.6999	–0.4142	0.2401
**Tree nuts**									
	BP	0.2521	0.6207	*–0.1732*	0.2178	0.0378	0.7432	0.0036	2.8525
	LN	0.2515	0.6210	*–0.1732*	0.2245	0.0412	0.7421	0.0065	3.1450
**Vegetal products**									
	BP	0.0517	0.8313	0.0030	0.1485	–18.9397	0.7823	0.7848	0.0053
	LN	0.0521	0.8214	0.0024	0.1521	–15.2981	0.7888	0.6954	0.0055
**Vegetable oils**									
	BP	0.0081	0.6667	–0.0007	0.1663	0.4467	0.5968	–0.0039	2.4944
	LN	0.0043	0.6821	–0.0014	0.1681	0.4428	0.5960	–0.0041	2.4912
**Vegetables**									
	BP	2.7153	0.6205	–0.1959	0.1099	1.6107	0.5935	0.1003	0.0184
	LN	2.7154	0.6650	–0.1991	0.1124	1.5459	0.6520	0.1000	0.0156
**Obesity**									
	BP	0.0042	0.5954	–0.0009	0.1716	0.0042	0.4986	–0.0009	4.1135
	LN	0.0041	0.5954	–0.0005	0.1712	0.0041	0.5101	–0.0009	4.1232

^a^BP: beta prime.

^b^Empty cells related to unestimated or nonconverged values.

^c^LN: log-normal.

^d^Italics indicate statistical significance at the .05 significance level.

According to the results of Table 1, except pulses in fat quantity and animal products, meat, tree nuts, and vegetables in the protein data set, all categories had no significant effect on the number of deaths. A 1% increase in supplementation of pulses reduced the odds of having a zero death by 4-fold (1 / exp(–1.417) = 4.1251). In addition, a 1% increase in supplementation of animal products and meat increased the odds of having a zero death by 1.076-fold (exp(0.0736) = 1.076) and 1.133-fold (exp(0.0736) = 1.133), respectively. Tree nuts reduced the odds of having a zero death, and vegetables increased the number of deaths.

**Figure 2:**
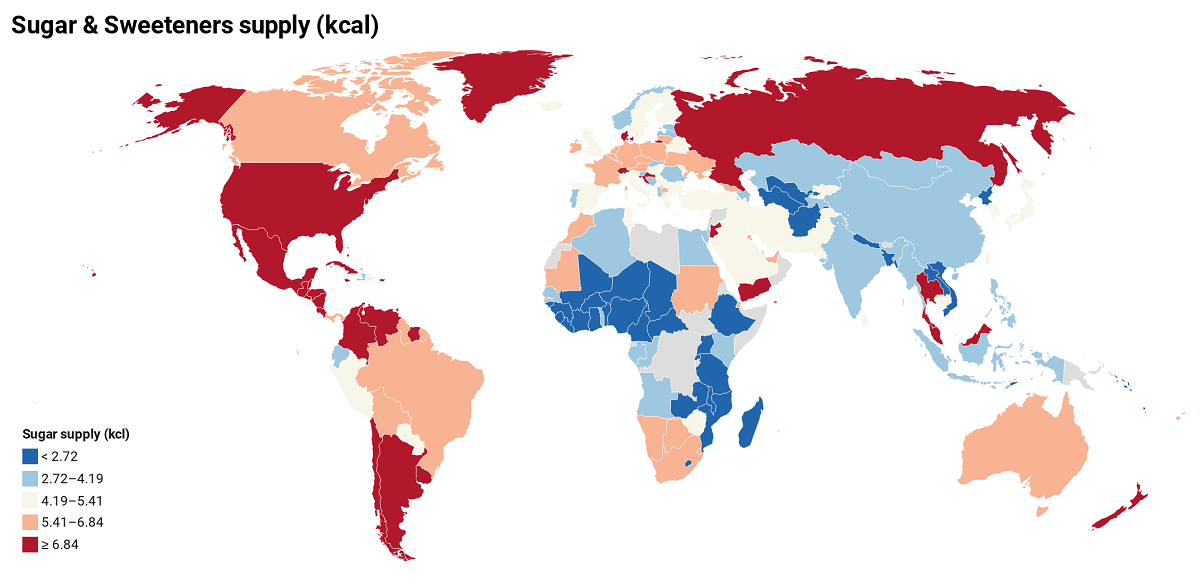
World map related to sugar and sweeteners supply (kcal).

Continuously, except animal fats, sugar sweeteners, and tree nuts in fat quantity, and animal fats and sugar crops in the protein data, all categories had no significant effect on the number of recoveries (Table 2). The effect of consuming sugar products on mortality was considerable. Every 1% increment in sugar sweeteners decreased the number of recoveries by 98.17% (–9.68, 95% CI –12.6440 to –6.7098). Tree nuts in fat quantity also reduced the number of recoveries by 16.9% (–0.1732, 95% CI –0.3157 to –0.3070). In the protein data, sugar crops reduced the number of recoveries by 99.11% (1 – exp(–4.7273) = 0.9911). The world map related to sugar and sweetener supply is shown in Figure 2. Based on the results of the proposed model and estimates of the effects of sugar, our prediction for the coming days is that the countries of the Americas, with more sugar product intake, will probably face more deaths.

**Figure 3:**
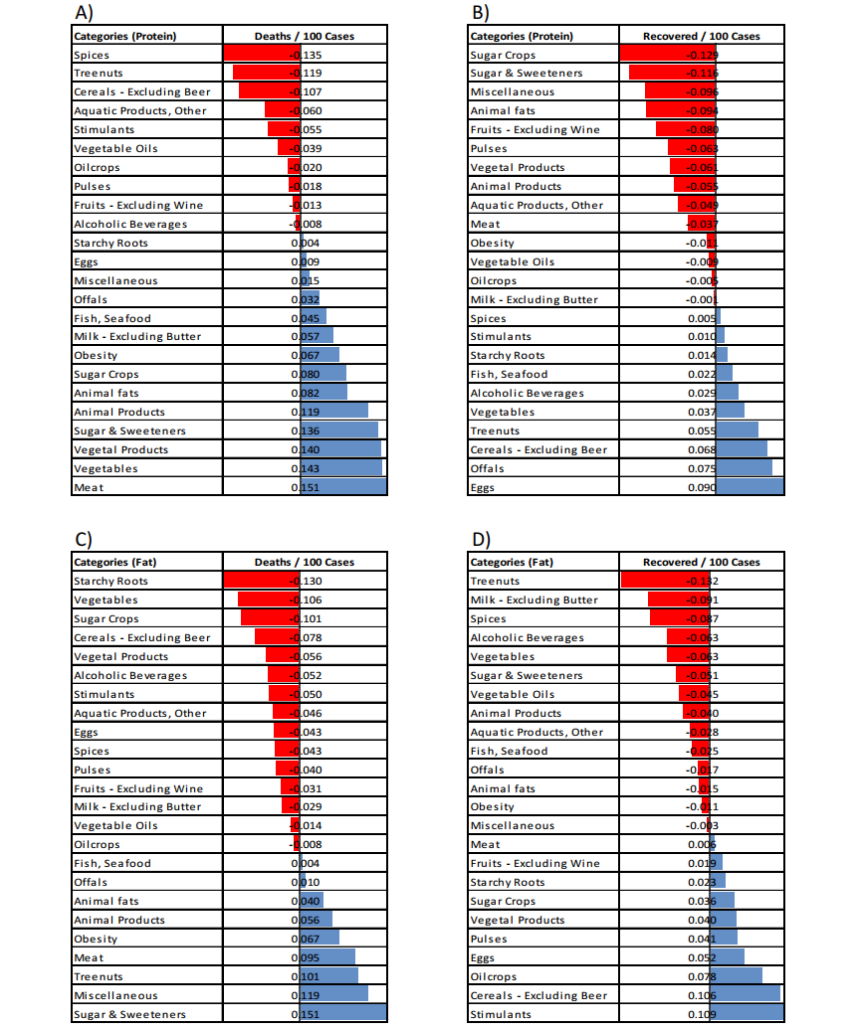
Bivariate Pearson correlation between nutrition categories (plus obesity) and the number of deaths (A and C) and the number of recoveries (B and D) in 100 cases of COVID-19.

**Figure 4:**
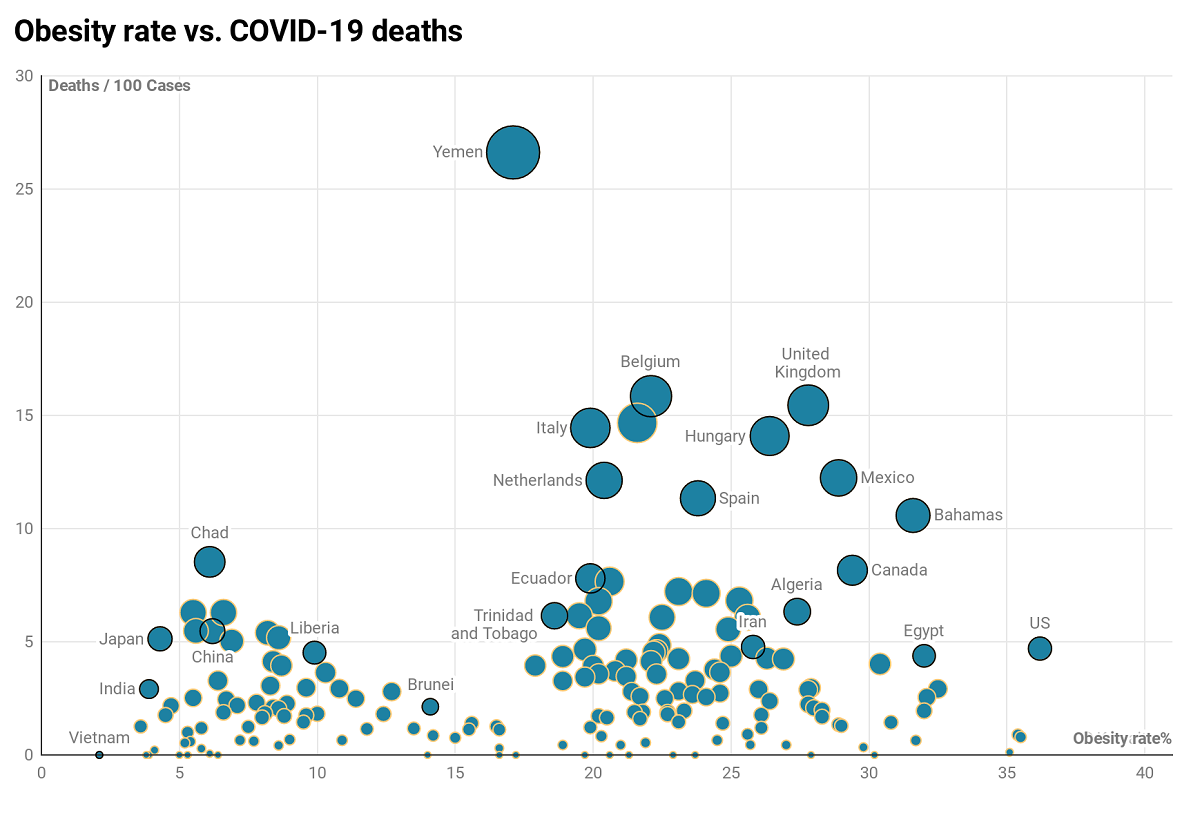
Scatterplot of obesity rate versus deaths per 100 cases of COVID-19 by country. The circle’s size is related to the number of deaths per 100 cases.

For further evaluation, we calculated correlations between categories (plus obesity rate) with the number of deaths (Figure 3 A and C) and the number of recoveries (Figure 3 B and D) by using the bivariate Pearson correlation. Results of the correlations showed that, in the protein data, countries that consumed more spices, tree nuts, cereals, aquatic products, stimulants, vegetable oils, oil crops, pulses, fruit (wine), and alcoholic beverage (in order) had fewer deaths from COVID-19, and conversely, countries that consumed more meat, vegetables, vegetal products, sugar and sweeteners, animal products, animal fats, sugar crops, milk, fish, offals, miscellaneous, eggs, and starchy roots (in order) had more deaths from COVID-19. In the fat quantity data, countries that consumed more sugar and sweeteners, miscellaneous, tree nuts, meat, animal products, animal fats, offals, and fish had more deaths from COVID-19. Finally, same as the mTP model results, obesity has affected increased death rates and reduced recovery rates in all correlation analyses (Figures 3 and 4).

## Discussion

### Principal Results

In this study, we proposed a mTP regression model for clustered semicontinuous diet and nutrition data. This model allows investigators to obtain covariate effects on the marginal mean of the outcome (eg, deaths and recoveries). It also has an unconditional interpretation of the covariate effect on the marginal mean. Our proposed mTP model had satisfactory performance in the diet and nutrition data analysis.

Findings of this study show that populations (countries) who consume more eggs, cereals excluding beer, spices, and stimulants had the greatest impact on the recovery of patients with COVID-19. In addition, populations that consumed more meat, vegetal products, sugar and sweeteners, sugar crops, animal fats, and animal products were associated with more deaths and less recoveries in patients. The effect of consuming sugar products on mortality was considerable. In addition, obesity has affected increased death rates and reduced recovery rates.

### Comparison With Prior Work

Healthy diets and physical activity are key to good nutrition and necessary for a long and healthy life and prevention of chronic disease [33]. Eating nutrition dense foods and balancing energy intake with necessary physical activity to maintain a healthy weight is essential at all stages of life. Unbalanced consumption of foods high in energy (sugar, starch, and fat) and low in essential nutrition contributes to energy excess, being overweight, and being obese. The amount of energy consumed in relation to physical activity and the quality of food are key determinants of nutrition-related chronic disease [11]. In a review study from January 2020, Zhang and Liu [13] reviewed the importance of some nutrition interventions (vitamins, minerals, immunoenhancers) in infectious and respiratory diseases. The authors suggested that the nutritional status of each patient who was infected should be evaluated before the administration of general treatments, and the current children’s RNA‐virus vaccines, including the influenza vaccine, should be used for people who are not infected and health care workers. Moreover, the results of their review showed that all the potential interventions (nutritional or immunoenhancers) should be implemented to control COVID‐19 if the infection is uncontrollable [13]. Our results also confirm these associations by introducing influential diet categories, including sugar and sweeteners, animal products, animal fats, sugar crops, miscellaneous, and tree nuts as more important risk factors for death or slowing of recovery in patients with COVID-19.

Recent studies point to obesity as a critical risk factor for being hospitalized or dying from COVID-19 [34-36]. Indeed, a high prevalence of obesity has been observed in patients with COVID-19, requiring invasive mechanical ventilation [37], a robust proxy of SARS-CoV-2 severity. In patients younger than 60 years, those with obesity were at almost double the risk of being admitted to critical care when compared with patients of a normal weight [38]. Results from this study confirm previous findings on the risk of obesity and add that obesity slows down patients’ recovery and treatment.

People need to eat fewer prepared foods and more complex plant-based foods [11]. Although there are differences in dietary patterns, overall, unbalanced diets are a health threat across the world and do not just affect death rates but also the quality of life. To achieve best results in preventing nutrition-related pandemic diseases, strategies and policies should fully recognize the essential role of both diet and obesity in determining good nutrition and optimal health. Policies and programs must address the need for change at the individual level as well as the modifications in society and the environment to make healthier choices accessible and preferable.

### Study Limitations

We have some limitation in using the nutrition data sets. The study is based on observational data, and inevitably with 188 countries included, there were variations in how the data were collected. This study included 23 dietary attributes; some that are of interest to health such as saturated and monounsaturated fatty acids and free sugars across the diet (not just those in drinks) were not included in the analysis. The study also did not take into account lifestyle factors, such as smoking and physical activity, that can have a significant impact on the risk of the disease outcomes used in the study.

Finally, we remind all our readers to take care of themselves during this pandemic, follow the guidelines of the Centers for Disease Control and Prevention [39], and eat healthy foods with sufficient amounts of fruits and vegetables as previously discussed.

### Conclusions

Good nutrition is important before, during, and after an infection. The findings of this study show that populations who consume more eggs, cereals excluding beer, spices, and stimulants had the greatest impact on the recovery of patients with COVID-19. In addition, populations that consumed more meat, vegetal products, sugar and sweeteners, sugar crops, animal fats, and animal products were associated with more deaths and less recoveries in patients. The effect of consuming sugar products on mortality is considerable. In addition, obesity has affected increased death rates and reduced recovery rates. Although there are differences in dietary patterns, overall, unbalanced diets are a health threat across the world and affect not only death rates but also the quality of life. To achieve best results in preventing nutrition-related pandemic diseases, strategies and policies should fully recognize the essential role of both diet and obesity in determining good nutrition and optimal health. Policies and programs must address the need for change at the individual level as well as the modifications in society and the environment to make healthier choices accessible and preferable.

## Appendix

Multimedia Appendix 1 Geographical distribution of the six World Health Organization regions and member states for each region (alphabetical order).

Multimedia Appendix 2 The specific types of food that belong to each category for the fat quantity and protein data sets.

Multimedia Appendix 3 Number of deaths per 100 cases, number of recoveries per 100 cases, and obesity rates until July 3, 2020, by countries and split by World Health Organization regions.

## Abbreviations

BP: beta prime
FAO: Food and Agriculture Organization
LN: log-normal
mTP: marginalized two-part
TP: two-part
WHO: World Health Organization
