# Supplementary figures and images for: Diet, Nutrition, Obesity, and Their Implications for COVID-19 Mortality: Development of a Marginalized Two-Part Model for Semicontinuous Data
Source: JMIR Public Health Surveill. 2021 Jan 26;7(1):e22717. doi: 10.2196/22717 (PMC7842860; doi:10.2196/22717)

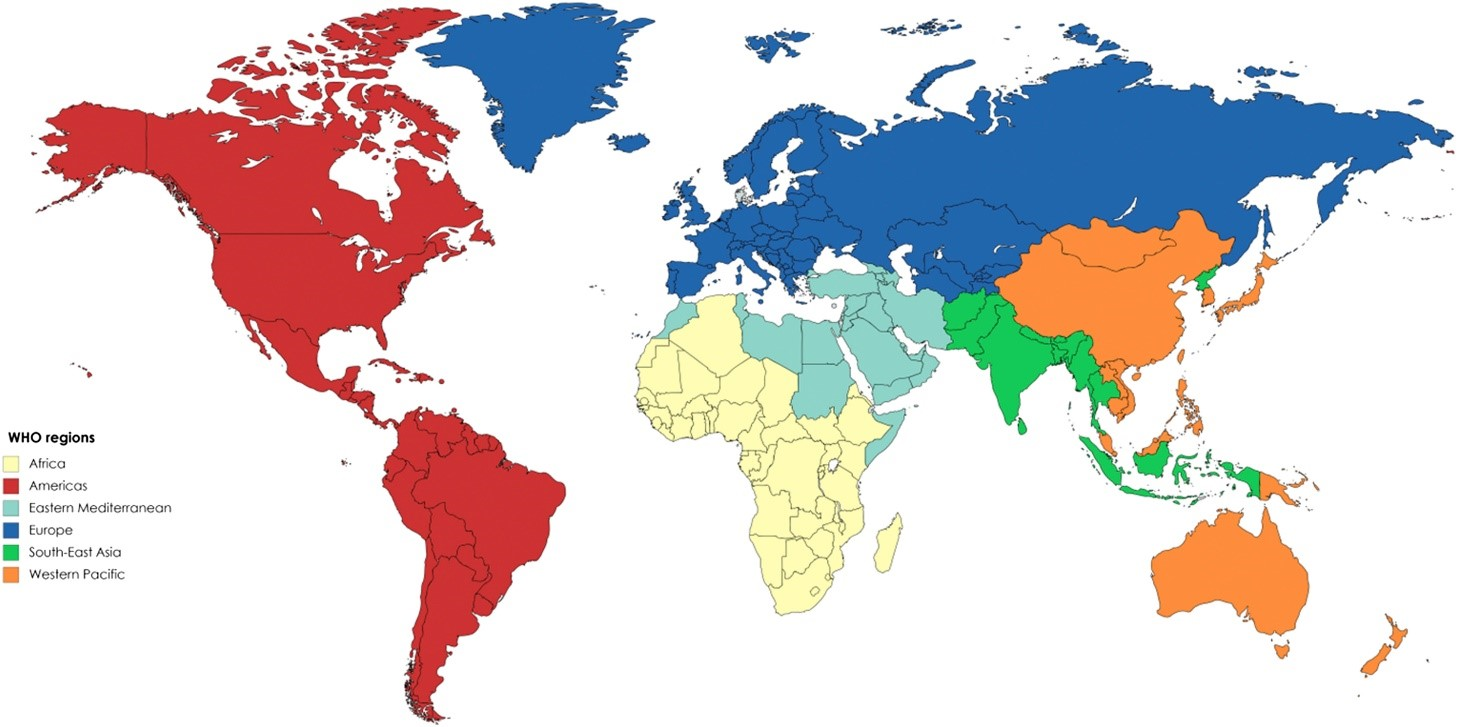

Supplement: Multimedia Appendix 1 [file publichealth_v7i1e22717_app1.png]

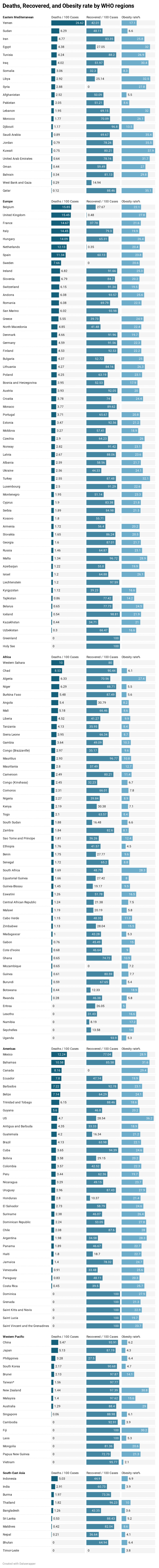


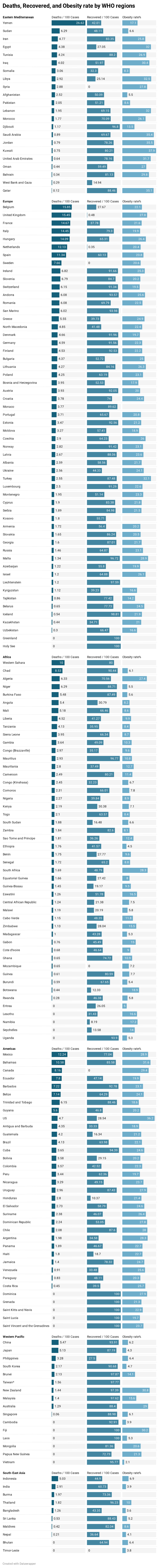


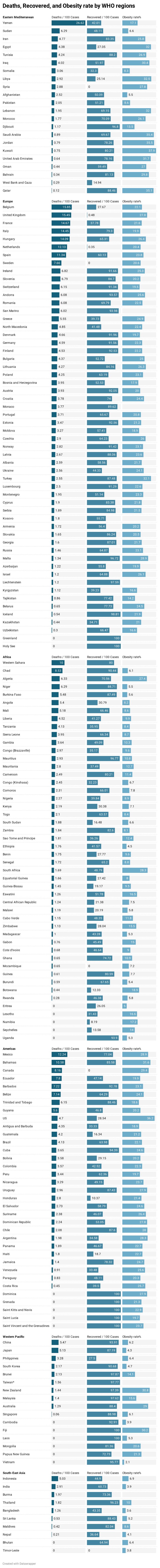


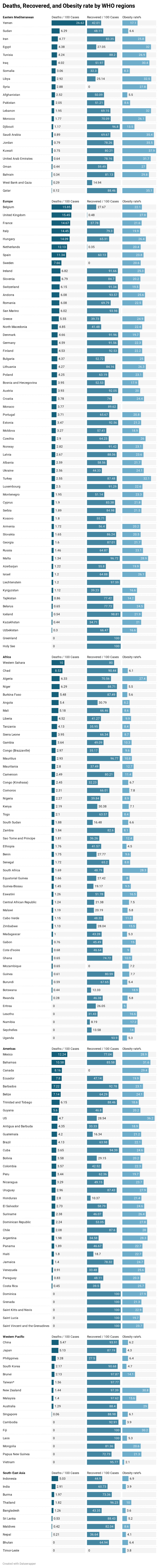

Supplement: Multimedia Appendix 3 [file publichealth_v7i1e22717_app3.docx]
