# Supplementary material for: Diet, Nutrition, Obesity, and Their Implications for COVID-19 Mortality: Development of a Marginalized Two-Part Model for Semicontinuous Data
Source: JMIR Public Health Surveill. 2021 Jan 26;7(1):e22717. doi: 10.2196/22717 (PMC7842860; doi:10.2196/22717)
Supplement: Multimedia Appendix 2 [file publichealth_v7i1e22717_app2.docx]

|  | **Items** |
| --- | --- |
|  |  |
| **Categories** |  |
| Alcoholic Beverages | Alcohol, Non-Food; Beer; Beverages, Alcoholic; Beverages, Fermented; Wine |
| Animal fats | Butter, Ghee; Cream; Fats, Animals, Raw; Fish, Body Oil; Fish, Liver Oil |
| Animal Products | Aquatic Animals, Others; Aquatic Plants; Bovine Meat; Butter, Ghee; Cephalopods; Cream; Crustaceans; Demersal Fish; Eggs; Fats, Animals, Raw; Fish, Body Oil; Fish, Liver Oil; Freshwater Fish; Marine Fish, Other; Meat, Aquatic Mammals; Meat, Other; Milk - Excluding Butter; Molluscs, Other; Mutton & Goat Meat; Offals, Edible; Pelagic Fish; Pigmeat; Poultry Meat |
| Aquatic Products, Other | Aquatic Animals, Others; Aquatic Plants; Meat, Aquatic Mammals |
| Cereals - Excluding Beer | Barley and products; Cereals, Other; Maize and products; Millet and products; Oats; Rice (Milled Equivalent); Rye and products; Sorghum and products; Wheat and products |
| Eggs | Eggs |
| Fish, Seafood | Cephalopods; Crustaceans; Demersal Fish; Freshwater Fish; Marine Fish, Other; Molluscs, Other; Pelagic Fish |
| Fruits - Excluding Wine | Apples and products; Bananas; Citrus, Other; Dates; Fruits, Other; Grapefruit and products; Grapes and products (excl wine); Lemons, Limes and products; Oranges, Mandarines; Pineapples and products; Plantains |
| Meat | Bovine Meat; Meat, Other; Mutton & Goat Meat; Pigmeat; Poultry Meat |
| Milk - Excluding Butter | Milk - Excluding Butter |
| Miscellaneous | Infant food; Miscellaneous |
| Offals | Offals, Edible |
| Oilcrops | Coconuts - Incl Copra; Cottonseed; Groundnuts (Shelled Eq); Oilcrops, Other; Olives (including preserved); Palm kernels; Rape and Mustardseed; Sesame seed; Soyabeans; Sunflower seed |
| Pulses | Beans; Peas; Pulses, Other and products |
| Spices | Cloves; Pepper; Pimento; Spices, Other |
| Starchy Roots | Cassava and products; Potatoes and products; Roots, Other; Sweet potatoes; Yams |
| Stimulants | Cocoa Beans and products; Coffee and products; Tea (including mate) |
| Sugar & Sweeteners | Honey; Sugar (Raw Equivalent); Sugar non-centrifugal; Sweeteners, Other |
| Sugar Crops | Sugar beet; Sugar cane |
| Treenuts | Nuts and products |
| Vegetable Oils | Coconut Oil; Cottonseed Oil; Groundnut Oil; Maize Germ Oil; Oilcrops Oil, Other; Olive Oil; Palm Oil; Palmkernel Oil; Rape and Mustard Oil; Ricebran Oil; Sesameseed Oil; Soyabean Oil; Sunflowerseed Oil |
| Vegetables | Onions; Tomatoes and products; Vegetables, Other |
| Vegetal Products | Alcohol, Non-Food; Apples and products; Bananas; Barley and products; Beans; Beer; Beverages, Alcoholic; Beverages, Fermented; Cassava and products; Cereals, Other; Citrus, Other; Cloves; Cocoa Beans and products; Coconut Oil; Coconuts - Incl Copra; Coffee and products; Cottonseed; Cottonseed Oil; Dates; Fruits, Other; Grapefruit and products; Grapes and products (excl wine); Groundnut Oil; Groundnuts (Shelled Eq); Honey; Infant food; Lemons, Limes and products; Maize and products; Maize Germ Oil; Millet and products; Miscellaneous; Nuts and products; Oats; Oilcrops Oil, Other; Oilcrops, Other; Olive Oil; Olives (including preserved); Onions; Oranges, Mandarines; Palm kernels; Palm Oil; Palmkernel Oil; Peas; Pepper; Pimento; Pineapples and products; Plantains; Potatoes and products; Pulses, Other and products; Rape and Mustard Oil; Rape and Mustardseed; Rice (Milled Equivalent); Ricebran Oil; Roots, Other; Rye and products; Sesame seed; Sesameseed Oil; Sorghum and products; Soyabean Oil; Soyabeans; Spices, Other; Sugar (Raw Equivalent); Sugar beet; Sugar cane; Sugar non-centrifugal; Sunflower seed; Sunflowerseed Oil; Sweet potatoes; Sweeteners, Other; Tea (including mate); Tomatoes and products; Vegetables, Other; Wheat and products; Wine; Yams |
